# Anatomisation with slicing: a new privacy preservation approach for multiple sensitive attributes

**DOI:** 10.1186/s40064-016-2490-0

**Published:** 2016-07-04

**Authors:** V. Shyamala Susan, T. Christopher

**Affiliations:** PG and Research Department of Computer Science, Government Arts College, Udumalpet, India; PG and Research Department of Computer Science, Government Arts College, Coimbatore, India

**Keywords:** Privacy preservation, Anatomization, Slicing, k-Anonymity, *l*-Diversity

## Abstract

An enormous quantity of personal health information is available in recent decades and tampering of any part of this information imposes a great risk to the health care field. Existing anonymization methods are only apt for single sensitive and low dimensional data to keep up with privacy specifically like generalization and bucketization. In this paper, an anonymization technique is proposed that is a combination of the benefits of anatomization, and enhanced slicing approach adhering to the principle of k-anonymity and *l*-diversity for the purpose of dealing with high dimensional data along with multiple sensitive data. The anatomization approach dissociates the correlation observed between the quasi identifier attributes and sensitive attributes (SA) and yields two separate tables with non-overlapping attributes. In the enhanced slicing algorithm, vertical partitioning does the grouping of the correlated SA in ST together and thereby minimizes the dimensionality by employing the advanced clustering algorithm. In order to get the optimal size of buckets, tuple partitioning is conducted by MFA. The experimental outcomes indicate that the proposed method can preserve privacy of data with numerous SA. The anatomization approach minimizes the loss of information and slicing algorithm helps in the preservation of correlation and utility which in turn results in reducing the data dimensionality and information loss. The advanced clustering algorithms prove its efficiency by minimizing the time and complexity. Furthermore, this work sticks to the principle of k-anonymity, *l*-diversity and thus avoids privacy threats like membership, identity and attributes disclosure.

## Introduction

Today’s health care providers store, and transmit a huge amount of sensitive data as a content of their business. The sensitive data can be personally recognizable information from the clients. Any kind of misuse of this information creates a critical threat to their business. When making the sensitive data available to the public, it is necessary for them to protect it from any abuse.

From a data privacy protection point of view, data anonymization is the one and only popularly used approach. It modifies information, keeping in mind to make it difficult to link individuals with their data. This methodology tries to ensure the identity along with the sensitive information of the data subjects when data is shared for diverse purposes (LeFevre et al. 2008; Aggarwal et al. 2005; Pfitzmann and Hansen 2008). SA is the set of attributes whose values are confidential such as cancer type, treatment, symptom, date of diagnosis and physician. The other attributes are related to the identifiers whose values assist in distinctly performing the identification of an individual like name or id, and QI attributes are those attributes which help in recognizing an individual when collected together. These attributes may be considered with caution so that there exists no leakage of information.

When sharing records, it is very important to avoid the disclosure of sensitive information of the individuals. There are three basic privacy disclosures that have been so far identified. They are identity disclosure, membership disclosure and also attribute disclosure. Identity disclosure happens when the character is linked to a specific record inside the shared data set. Attribute disclosure happens when the new approximate information about some man or woman is found out, which in turn indicates that the shared data render it feasible to be able to retrieve the individual’s characteristics with greater certainty than could be got earlier with the records shared. Membership disclosure happens when the information disclosed is about whether the record of an individual exists in the data that is published or not.

There are multiple anonymization methods that prevail for retaining privacy. They are namely generalization, suppression, anatomization, bucketization, permutation, and perturbation. Generalization and suppression concentrate on QI attributes, whereas bucketization is focused on splitting SA from QI attributes with a description that is less specific. Anatomization and permutation dissociate the correlation between QI attributes and SA the by collection and rearrangement of sensitive values in a *qid* group. Perturbation tampers the data by the addition of noise, aggregation of values, swapping of values, or generation of artificial data or by the encryption of the data, in the light of few measurable characteristics of the first information. Slicing is a technique that can tackle with high dimensional data and hence preserve privacy and improve utility.

The majority of the strategies above focus on anonymizing the micro data with only single SA. As they are not suitable for functional usage, the current challenge is to preserve the multiple SA efficiently in the high dimensional data.

### Motivation

The generalization for k-anonymity (Gedik and Liu 2008; El Emam and Dankar 2008) and bucketization for *l*-diversity (Machanavajjhala et al. 2007; Ninja et al. 2007) are popularly understanding privacy preservation strategies. Generalization for k-anonymity (Aggarwal 2005; Gehrke 2006) ignores a huge measure of data in case they are high dimensional data.

In order to get over deformities in generalization, an inventive anatomization methodology is brought into use (Xiao and Tao 2006). It reduces the data loss, although it is capable of preserving privacy for single sensitive data only. The anatomization preserves privacy as it is not representative of the sensitive value corresponding to any tuple, which might be assumed randomly from ST. A larger *l* indicates more privacy. So taking the significance of both individual’s privacy and utility into consideration, an algorithm, called as kl-redInfo, is proposed which enhances the anatomy algorithm. This is performed by presenting new approaches with the systematic integration of the remaining records, cell-based generalization in place of separation of the table into two parts, and sorting the records as per their QI attributes for the purpose of reducing the total amount of information loss.

In order to ensure privacy for high dimensional data, a new slicing methodology (Li et al. 2012) is utilized. This helps in preserving preferable data utility than generalization and also preserves correlation. This system, in addition, yields support to single sensitive data only. For the purpose of handling privacy for several SA a multiple sensitive bucketization (MSB) (Yang et al. 2008) is introduced. But this method is appropriate for less than three SA only. For greater SA slicing with MSBKACA (Han et al. 2013) is employed. As it renders support to generalization its execution time is high and there is a significant amount of impressive measure of data loss.

Motivated by means of these works, this paper focuses on the preservation of the privacy of data with numerous SA with lesser information loss and better data utility. In this work an anatomization approach is employed to minimize the information loss by releasing the QI attributes directly. On the contrary, slicing maintains the correlation in the column and then carries out the break of correlation across the columns by means of vertical and horizontal partitioning. In order to preserve the correlations existing between the attributes, it groups the highly-correlated attributes together. Slicing does the permutation of the sensitive attributes within each bucket in order to carry on the correlation break across the columns, and assures privacy. Every attribute in a column can be considered in the form of a sub table. This removes the dimensionality with respect to the data. Additionally, the research work functions in accordance with the principle of k-anonymity and *l*-diversity that does not impact the QI values which are directly released by means of anatomization. This provides the way for preventing membership, identity and attributes disclosures. The advanced clustering algorithms that are included in slicing showed their significance through the minimization of the time and complexity. Empirical evaluation of the actual health care data related to the Cleveland heart disease dataset and Hungarian Institute of Cardiology proves the resourcefulness of this model.

### Contribution

This proposed approach integrates the benefits of both anatomisation and enhanced slicing algorithm adhering to the principle of *l*-diversity and k-anonymity and hence deals with the difficulty in conducting the multiple SA in high dimensional data. For the exemplification of the proposed approach, Table 1 having six SA is considered. The attributes presented in the table are Patient-Id, Gender, Zip code, Age, Cancer type, Treatment, Symptom, Date of diagnosis, Physician, Diagnosis method. Out of these attributes, Cancer type, Treatment, Symptom, Date of diagnosis, Physician and Diagnosis method are the SA. Alternatively, QI attributes include Patient-Id, Gender, Zip code, Age. At first, the proposed approach anatomizes Table 1 by dissociating the QI attributes from SA and provides two tables, one for the QI attributes and the other for the SA. The results are shown in Tables 2 and 3.

**Table 1:**
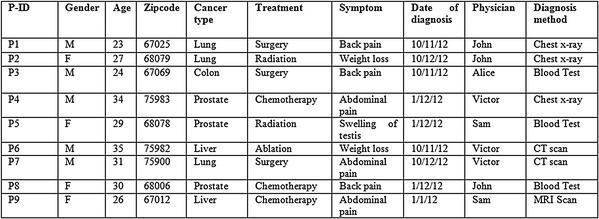
Original table

**Table 2:**
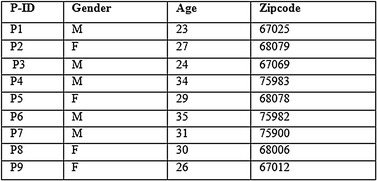
Quasi table (QIT)

**Table 3:**
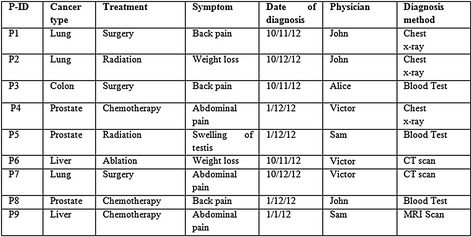
Sensitive attribute table (ST)

This is subsequently followed by employing a slicing technique to Tables 2 and 3. This segregates the tables both horizontally and vertically. In the vertical partitioning phase, the advanced clustering algorithm is applied to the Table 3 and the outcome of this step are highly correlated attributes are in one column. For example {Cancer type, Treatment}, {Symptom, Date of diagnosis}, {Physician, Diagnosis method} are the correlated attributes. Thus, Table 3 is partitioned into three different SA tables. In a similar way the QI attributes in Table 2 are partitioned such that highly correlated attributes are in one column. For example {{Gender, Age}, {Zip code}}.

The horizontal partitioning phase is referred to as tuple partitioning. In this phase, tuples in each of the 3 ST are bucketised making use of MFA. In order to attain *l*-diversity, attributes in each bucket are chosen as a distinct one. As the tuple imposes 3 diversity, each bucket also imposes 3 different attributes and this is seen in Tables 4, 5 and 6. For example in Disease column, the different SA {Lung, Prostate, Liver} are in bucket 1, {Lung, Colon, Prostate} are in bucket 2 and {Prostate, Lung, Liver} are in bucket 3.

**Table 4:**
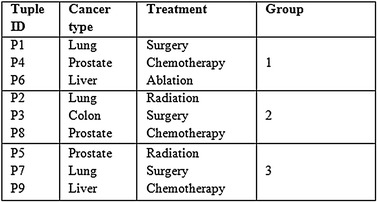
Sliced sensitive attributes (cancer—treatment)

**Table 5:**
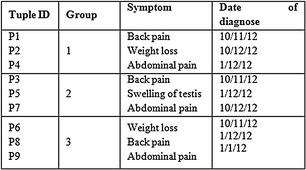
Sliced sensitive attributes (symptom—date)

**Table 6:**
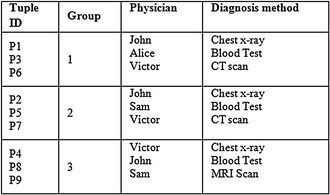
Sliced sensitive attributes (physician—diagnosis method)

Similarly, the tuples in QIT are bucketised making use of MFA. In order to attain 3 anonymity, for all the individuals in Table 2, there are at least 3 individuals that are connected to the same bucket of sensitive values. The bucket imposes 3 different attributes, and this is seen in Table 7. For example, in {Age, Sex} column the different attributes are {(23, M)}, {(24, M)}, {(26, F)}. All the tables from Tables 4, 5, 6 and 7 have a common column referred to as group Id for linking. This Id does the mapping of the QIT with the multiple ST. In this way, the horizontal and vertical partitioning aids in the elimination of the dimensionality of the dataset.

**Table 7:**
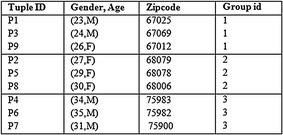
Sliced quasi identifier attributes

Within every bucket, the values in every column are permutated randomly for breaking the connection between various columns. For example, in the first bucket of the sliced table as indicated in Table 8 the values are then permutated stochastically in such a manner that the linkage observed between the two columns in one bucket gets hidden. This is observed in Table 8. This feature therefore makes it possible to publish all the data available in a single table, and still the privacy is preserved. In this way the slicing procedure assures that in the case of any tuple, there exists as many matching buckets.

**Table 8:**
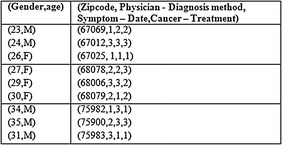
Anonymised data

The research work functions in accordance with the principle of k-anonymity and *l*-diversity that does not impact the QI values which are directly released by means of anatomization. This results in preventing membership, identity and attributes disclosures. Resultant Table 8 is considered for the purpose of demonstrating the process.

Consider the patient Id P2 with QI values (f, 27, and 68079). In order to decide P2’s multiple sensitive values, P2’s matching bucket has to be decided. By examining (f, 27), it is a known fact that P2 should be present in bucket 2, as there seems to have no matches in bucket 1 and bucket 3. Then by examining the Zip code attribute in bucket 2, the matching value is (68079, 2, 1, 2). This indicates that {Physician—Diagnosis} method points to bucket 2 of Table 6, {Symptom—Date} points to bucket 1 of Table 5 and {Cancer—Treatment} points to bucket 2 of Table 4. It is inferred that (f, 27, 68079) may have different values like{John, Sam, Victor} for Physician and,{Chest X-ray, Blood Test, CT scan}for diagnosis method, {Back pain, Weight loss, Abdominal pain}for Symptom, {10/11/12, 10/12/12, 1/12/12} for date of diagnosis and {Lung, Colon, Prostate}for cancer type and {Radiation, Surgery, Chemotherapy} for treatment. Thus, it results in 3 anonymity, because when an individual is mapped onto some sensitive value, at least 2 other individuals are also mapped to the same sensitive values. And it satisfies 3 diversity because it poses 3 distinct sensitive values in each bucket. Thus release of the QI values preserve privacy such that the sensitive value pertaining to an individual that is involved in the QIT can be rightly guessed by an intruder with the computed probability of at most 1/3.

The anatomization approach reduces the loss of information through the direct release of the QI attributes. In the slicing technique, vertical partitioning does the grouping of the correlated SA in ST together and thereby minimizes the dimensionality by employing the advanced clustering algorithm (ACA). In order to get the optimal size of buckets, tuple partitioning is conducted by MFA. Thus the advanced clustering algorithms reduce the complexity. Membership and identity disclosures are preserved by k-anonymity and attribute disclosures are eliminated by *l*-diversity.

## Background

Privacy preserving data mining (PPDM) is a rapidly growing research area aiming at eliminating privacy breaches which may happen during the mining of data (Verykios et al. 2004; Kantarcioglu et al. 2004; Clifton 2009). The goal of PPDM algorithm is to alter the original data for the purpose of maintaining privacy, leading to a low degree of data leakage. This will give way for obtaining good mining results. The work introduced in (Verykios et al. 2004) observes the PPDM approach in the light of five different dimensions. They are data distribution, data modification, data mining algorithm, data or rule hiding and privacy preservation. Data distribution represents the organization of data that can be centralized or in a distributed fashion. The second step data modification refers to modifying the data.

The work introduced in (Friedman et al. 2008) yields the possibilities for the construction of k-anonymous data models with k-anonymous data sets. More commonly, the k-anonymity concept is utilized by the PPDM algorithms in order to guarantee privacy (El Emam and Dankar 2008). It is a problem to be able to find optimal k-anonymous datasets through generalization and is rated as NP-Hard (Gedik and Liu 2008; Meyerson and Williams 2004). The work indicated in (Li and Li 2006) compares the general taxonomy and multiple generalization schemes. The work that is shown in (Iyengar 2002) formulated a genetic framework in order to look for the best set of generalization for the purpose of satisfying k-anonymity constraints. Hence, each generalization is considered a chromosome.

In Wang et al. (2004), a work providing privacy by a bottom-up generalization scheme is presented. The work provided in Fung et al. (2007) introduces a generalization method for classification through the application of k-anonymity and it is a top down specialization algorithm. This algorithm is rather better than the bottom-up approach. In Nergiz and Clifton (2007), the authors made enhancement to the algorithm that is presented in Fung et al. (2007). Many clustering techniques are illustrated in Mandapati et al. (2013) for the generation of domain hierarchies.

The work as shown in Karthikeyan et al. (2011) collaborates that fuzzy logic is utilized for preserving sensitive information. At first, the dataset is clustered and then by making use of a fuzzy membership function, addition of noise takes place. A hybrid evolutionary algorithm that makes use of Genetic Algorithm and Particle Swarm Optimization is presented in Freitas (2005). Genetic algorithms are very much helpful in feature selection, during the mining of data (Gibbs et al. 2008; Zhang et al. 2009; Rokach 2008). A genetic algorithm based framework is introduced in Pham and Karaboga (2012) to resolve feature set partitioning tasks.

Anatomization (Xiao and Tao 2006) in contrast to generalization and suppression does not make modifications to the QI or the SA, but rather dissociates the relationship between the two. To be precise, the method gives out the data on QI and the data on the SA in two separate tables: a QIT containing the QI attributes, a ST containing the SA, and both QIT and ST has one common attribute, which is the group ID. The greatest benefit of anatomy is that there is no modification of data in both QIT and ST. Xiao and Tao proved that the anatomized tables can answer aggregate queries dealing with domain values of the QI and SA more accurately compared to the generalization approach. Tao et al. (2009) proposed an approach referred to as a permutation, sharing the same kind of spirit of anatomization. The point behind is to make the dissociation of the relationship between a QI and a numerical SA by the partitioning of a set of data records into groups and then shuffling their sensitive values within each group.

A new slicing (Li et al. 2012) approach is employed, which partitions the attributes both horizontally and vertically and avoids membership disclosure. Slicing with Modified Fully Self Adaptive Resonance Neural Network and Metaheuristic Fireflies with Minkowsi Distance Measure (Shyamala and Christopher 2015) improved the clustering accuracy in privacy preservation. But all of the above methods suit single sensitive data only. In order to deal with multiple SA a multiple sensitive bucketization (MSB) (Yang et al. 2008) is suggested. But it is appropriate for attributes less than three only. In case of more SA slicing with MSB.KACA (Han et al. 2013) is brought into use. As it follows generalization its execution time is high and there is a significant amount of quite a measure of data loss.

Inspired by these works, this paper aims at preserving the privacy of data with multiple SA. In this paper, an anonymization technique is proposed that is a combination of the benefits of anatomization, and enhanced slicing approach adhering to the principle of k-anonymity and *l*-diversity for the purpose of dealing with high dimensional data along with multiple sensitive data. The anatomization approach dissociates the correlation observed between the quasi identifier (QI) attributes and sensitive attributes (SA) and yields two separate tables with non-overlapping attributes. The enhanced slicing algorithm retains privacy through horizontal and vertical partitioning. In the vertical partitioning phase, the relevant SA are clustered by employing an advanced clustering algorithm (ACA) and gives rise to several sensitive tables (ST) having its group membership added in a new column group ID. In the subsequent phase the metaheuristic firefly algorithm (MFA) assembles the tuples into buckets in a horizontal manner and assures *l*-diversity in each ST. In the same manner, the QI attributes are divided under k-anonymity and then the new quasi identifier table (QIT) has the precise QI attribute values in addition to its group membership getting appended in a new column group ID. Finally, the SA in each group is shuffled and their linking with QI attributes in QIT is done with a common group ID. This ensures that the sensitive value pertaining to an individual could be inferred by means of an intruder with the chance of at the most 1/*l*. The experimental outcomes indicate that this method can preserve privacy of data with numerous SA. The anatomization approach minimizes the loss of information and slicing algorithm helps in the preservation of correlation and utility and this limits the information loss and helps in reducing the data dimensionality. The advanced clustering algorithms prove its efficiency by minimizing the time and complexity. Furthermore, this work sticks to the principle of k-anonymity, *l*-diversity and thus avoids privacy threats like membership, identity and attributes disclosure.

## Preliminaries

### Formalization of anatomy with slicing

Consider T to be a micro data with n QI attributes and m SA $\{ q_{1} , \ldots ,q_{n} ,s_{1} , \ldots ,s_{m} \}$.

### Definition: Anatomization

Anatomy dissociates the correlation between QI attributes with SA and generates QIT and ST. QIT has the dataset $(q_{1} ,q_{2} , \ldots ,q_{n} )$ and ST has the dataset $(s_{1} ,s_{2} , \ldots ,s_{m} )$.

The idea behind the anatomy approach is that in case two tables with a join attribute goes for publishing, then join corresponding to the two tables could be lossy and again this lossy join is useful in concealing the private data (Xiao and Tao 2006).

### Definition: k-Anonymity and *l*-diversity

With k-anonymity, it is required to be made sure that when an individual is mapped onto some sensitive values, at least k − 1 other individuals have their mapping to the same sensitive value. With *l*-diversity, it has to be assured that the diversity of the sensitive value is at least *l*.

### Attribute partition and column

Let ST be a dataset with m SA, $ST = \{ s_{1} ,s_{2} , \ldots ,s_{m} \}$ and their respective attribute domains $\{ D[s_{1} ]D[s_{2} ], \ldots ,D[s_{m} ]\}$ A tuple $t \in T$ can be denoted as $t = (t[s_{1} ],t[s_{2} ], \ldots ,t[s_{m} ])$ where $t[s_{i} ](1 \le i \le m)$, is the $s_{i}$ value of $t$.

An attribute partition contains different subsets of ST, in such a manner that each attribute belongs to only one subset. Each subset of attributes is called as a column. To be specific, let there be c columns $C_{1} ,C_{2} , \ldots ,C_{c}$, then $\bigcup\nolimits_{i = 1}^{C} {C_{i} } = A$ and for any $1 \le i{}_{1} \ne i{}_{2} \le C,C_{i1} \cap C_{i2} = \varphi$. Then each subset of attributes is generated as $ST_{1} ,ST_{2} , \ldots ,ST_{i}$ with the group Id. It contains the schema, (group Id, $s_{i}$) $(i = 1,2, \ldots ,m)$.

In a similar way, QIT has a dataset with n QI attributes.

$QIT = \{ q_{1} ,q_{2} , \ldots ,q_{n} \}$ and their attribute domains are represented $\{ D[q_{1} ], \ldots ,D[q_{n} ]\}$. A tuple t ∈ T can be denoted as $t = \left( {t[q_{1} ],t[q_{2} ], \ldots ,t[q_{n} ]} \right)$ where $t = t[q_{i} ]\left( {1 \le i \le n} \right)$ is the qi value of t. Each subset of attributes is referred to as a column. Particularly, let there be c columns $C_{1} ,C_{2} , \ldots ,C_{c}$ then ${\text{U}}_{i = 1}^{c} C_{i} = A$ and for any $1 \le i_{1} \ne i_{2} \le C,C_{i1} \cap C_{i2} = {\o}$. The QIT has the schema (group Id, $A_{1}^{{q_{1} }} ,A_{1}^{{q_{2} }} , \ldots ,A_{n}^{{q_{i} }}$).

### Tuple partition

A tuple partition comprises of different subsets of T, in such a way that every tuple belongs to only one subset exactly. Every subset consisting of tuples is referred to as a bucket. To be specific, consider b number of buckets $B_{1} , \ldots ,B_{b}$, then $\cup_{i = 1}^{b} B_{i} = T$ and thereafter for any $1 \le i_{1} \ne i_{2} \le b_{i} ,B{}_{i1} \cap B{}_{i2} = \varphi$.

### Privacy requirement

The privacy requirement required for publishing multiple sensitive data are k-anonymity and *l*-diversity. K-anonymity (El Emam and Dankar 2008), that helps in preventing the individual records identification in the data, and *l*-diversity (Machanavajjhala et al. 2006), which, on the other side, avoids the association of an individual record having a SA value.

In this approach slicing with k-anonymity guarantees that when an individual is mapped onto some sensitive values, at least k − 1 other individuals are also mapped onto the same sensitive values. In a similar way slicing based on *l*-diversity assures that the intruder shall not learn about the sensitive value with respect to any individual having the probability 1/*l*. Similarly, the privacy to the anonymised group G is extended. For each group G, the exact QID items are published in order to gather the group id of sensitive items.

The probability of association of the sensitive item in the anonymised group G is

1 $$Deg(G) = \min_{i = 1, \ldots ,m} |G|/f_{i}^{G}$$

The privacy degree of an entire partitioning $P$ of $T$ is

2 $$Deg(P) = \min_{G \in P} Deg(G)$$

### Utility requirements

In order to preserve the utility, grouping of highly-correlated attributes in one column are required. Slicing maintains the utility through the classification of the attributes that are correlated in one column and thereafter breaks the correlation which are seen across the columns. The published micro data are employed in order to obtain a particular pattern which can be expressed as a query. To decide on the utility of the anonymised group, the reconstruction error is measured. Patterns can be expressed as queries of the form

- SELECT COUNT (∗) FROM T
- WHERE (Sensitive Items are present)
- AND $$(q_{1} = val_{1} )\varLambda \cdots \varLambda (q_{r} = val_{r} )$$

The process of estimation of the result of the query for each anonymized group *G* is known as data reconstruction. The number of events of item *s* in *G is* denoted by *a*, and the number of tuples matching the QID selection by *b*. Then the estimated result of the query becomes *a* *·* *b/|G|.* In case of all tuples in *G* having identical QID, then either *b* = *|G|* or *b* = 0, and the reconstruction error is 0. Considering ideally, to reduce reconstruction error, *|G|* − *b* has to be minimized, hence in each group the QI attributes with minimum distances has to be included.

A much meaningful method of modeling such queries involving sensitive items is to make use of a probability distribution function *(PDF)* of an item $s \in S$ over the space that is defined by a number of *r* items in *Q.* If the query to be evaluated is inclusive of *r* QID items, then the total number of cells is 2*r*, that corresponds to all combinations in case of an item that is or is not present in a tuple that is having a “group-by” query on items *q*_1_*… q*_*n*_. The original PDF value of sensitive item *s* for a cell *C* is

3 $$Act_{C}^{s} = \frac{Occurrnecs\;of\;\sin \;C}{Total\;occurences\;of\;\sin \;T}$$

The estimated PDF, *Est*_*c*_^*s*^ is calculated in a similar manner, except that the numerator comprises *a* *·* *b*/*|G|* that is summed over all groups intersecting cell *C*. The utility obtained from the anonymized data is determined as the distance between the actual and estimated pdf over all cells, measured by *KL*-*divergence*, which is already inferred (Aggarwal 2005) as a purposeful metric for assessing the amount of information loss that is incurred by data anonymization:

4 $$KL_{divergence} (Act^{s} ,Est^{s} ) = \sum\limits_{\forall cellC} {Act_{C}^{s} } \log \frac{{Act_{C}^{s} }}{{Est_{C}^{s} }}$$

If $Act^{s}$ is identical to $Est^{s}$, $KL_{divergence} = 0$. Hence it is desired to determine the partition which loses the privacy degree p with a minimum reconstruction error.

### Privacy threats

While publishing the micro data there exists multiple privacy threats that have to be accomplished for the data to be considered are protected. In the case of an Identity disclosure threat, an individual is provided with a link to a particular record in the data which is divulged. Attribute disclosure, then publishes the sensitive attribute data regarding an individual and then the Membership disclosure also reveals the information regarding whether an individual’s record is present in the published data or not.

## Proposed work

### Anatomizing the dataset D

In order to defeat the defects of generalization, an inventive anatomization technique to attain privacy is presented which yields precise QI values. This phase dissociates the QI and SA in micro table T and produces two tables referred to as, QIT and ST. The QI attributes and SA have no overlap as the SA generally is not seen on publicly available datasets.

### Enhanced slicing algorithm

The slicing algorithm achieves preservation of privacy through horizontal and vertical partitioning. As this work focuses on multiple SA, SA that are related are grouped together based on their correlation. At that juncture SA are sufficiently clustered and results in different tables of SA making use of advanced clustering algorithm. And in the subsequent phase tuples are partitioned horizontally by means of MFA and *l*-diversity is checked in for each sensitive tuple. Every ST inserts the correlated attributes along with its group membership within a new column group ID. In the same way, the partitioning of QI is done under k-anonymity and then the new QIT contains all of its exact QI values along with its group membership within a new column group ID. The partitioning technique removes the dimensionality of the data that ensures this work to be able to deal with any number of sensitive attributes. Finally, the SA in each group is shuffled and thereafter linked with a common group id, in such a manner that the sensitive value corresponding to an individual can be found by an intruder with the probability of at the most 1/*l*. A larger *l* leads to a much stronger privacy.

#### Attribute partitioning (vertical)

The important goal of vertical partitioning is grouping correlated SA together in ST. This is helpful for both privacy and utility. Slicing helps in the preservation of utility as it does the grouping of highly-correlated attributes with one another, and this way assists in the preservation of the correlations that are present between such types of attributes. Slicing provides protection in such a manner that it breaks apart the associations existing between uncorrelated attributes that are infrequent and in this juncture, distinguishing.

At first, the data points (sensitive attributes) are distributed in the data space. The clustering algorithm is applied for grouping relevant SA and thus reduces dimensionality. The standard k-means (Machanavajjhala et al. 2006), k-medoid (Jain 2010) and PAM (Han et al. 2011) clustering algorithm in slicing (Li et al. 2012) is affected by the huge computational complexity for large databases and does not yield high quality cluster for high dimensional data. Here a new advanced clustering algorithm (ACA) (Susan and Christopher 2016) for partitioning the attributes into columns is introduced that can effectively help in improving the clustering speed and hence minimize the complexity involved in computation. It maintains two data structures, one for holding the attributes of clusters and the other for holding the minimum distance between the attributes in such a way that it can be utilized as a part of the next subsequent cycle. Pearson correlation coefficient gives a measure of the correlation between the input terms. It is a popular similarity measure (Lin et al. 2014). In this work, SA$\{ s_{1} , \ldots ,s_{m} \}$ are given as input. The correlation between the SA is calculated by

5 $$\gamma_{{s_{1} ,s_{2} }} = \frac{{m\sum\nolimits_{ct = 1}^{m} {w_{{ct,s{}_{1}}} \times w_{{ct,s_{2} }} } }}{{\left[ {\sqrt {m\sum\nolimits_{ct = 1}^{m} {w_{{ct,s_{1} }}^{2} + w_{{ct,s_{2} }}^{2} } } } \right]}}$$

Equation 1 calculates the correlation between all SA. The value ranges between −1 to 1. $w_{{ct,s{}_{1}}}$ and $w_{{ct,s_{2} }}$ are the co-occurrence of terms in the data. Hence, the value ranges between $- 1 \le \gamma_{{s_{1} ,s_{2} }} \le 1$. The distance between two SA is given by $dist_{{s_{1} ,s_{2} }} = 1 - \gamma_{{s_{1} ,s_{2} }}$

6 $$dist_{{s_{1} ,s_{2} }} = 1 - \gamma_{{s_{1} ,s_{2} }}$$

The distance measure computes the degree of correlation between two SA. In the case of $dist_{{s_{1} ,s_{2} }}$ yielding a smaller value, then SA s_1_ and s_2_ are related to one another. This way, the distance value has an inversely proportional relation with the correlation between the SA.

If the distance is minimum or equivalent, then the attribute will remain in its group which was allotted to it in the earlier cycle. By this way, there is no reason to compute the distance from this attribute cluster to the next k − 1 clusters, thus minimizing the processing time till the k − 1 group focuses. Else, the separation from the present cluster to all k clusters is to be computed and the closest cluster has to be located. This can improve the speed of clustering efficiently and minimize the computational complexity. This process goes until the stop criterion is attained. This provides new ST with correlated SA in one column and uncorrelated attributes across the column and thus releases many ST with its group membership in a new column group ID. The following algorithm is the vertical partitioning phase of the multiple SA. Repeat the step 3 for the case m = 1 to N. Then recalculate the new cluster center of the cluster until the total number of clusters.


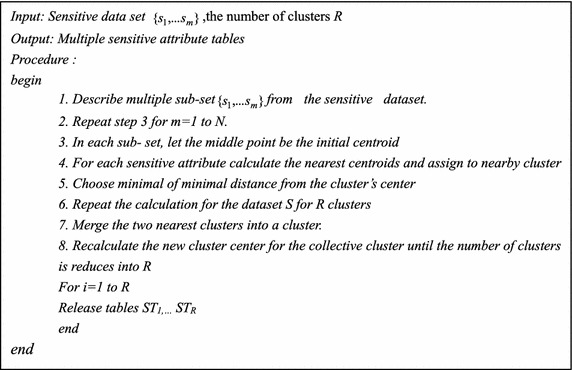


In a similar way related QI attributes are clustered and its dimensionality is minimized. This provides a new QIT containing correlated attributes in one column and uncorrelated attributes across the column with group membership in a new column group ID.

#### Tuple partitioning (horizontal)

Tuple partitioning is the process of horizontally grouping a table. Each horizontal group is referred to as buckets and the records are grouped in the respective buckets, thus satisfying *l*-diversity and k-anonymity. In this work, MFA (Arora and Singh 2013) with Minkowsi distance measure (MFAMD) is employed for appreciable grouping of records into buckets.

This algorithm is applied to QIT and each ST. The input to the QIT is QI attributes along with the k parameter and the input to the ST is SA along with *l* parameter. This algorithm horizontally partitions the dataset based on the size of the bucket. It categorizes the record by taking the different combinations of the SA and QI attributes into account. K-anonymity is then checked for QIT and *l*-diversity is checked for each of the ST. For instance, consider the SA, Cancer—Treatment. As assumed, horizontal partitioning must be performed in such a way that all the buckets possess different cancer type and treatment methods. This makes sense in a way that different combinations of Cancer—Treatment, should contain in each bucket. This is accomplished by MFA (Arora and Singh 2013) with MFAMD algorithm.

FA possesses two considerable benefits in comparison to other algorithms: First this algorithm does the grouping of attributes in the tuples on the basis of two parameters namely the light intensity and attractiveness. The light intensity value is decided by means of the objective function that helps in the subdivision of the tuples into subgroups with minimum number of tuples. Secondly, this subdivision permits the fireflies to be capable of finding all optima simultaneously in case the tuple size is sufficiently higher compared to the number of attributes in the group.

The average distance between the group of fireflies is controlled by 1/√γ. While γ = 0, the fireflies will not form a group. This feature is appropriate for high dimensional data to be categorized into several groups. The randomness characteristic of the firefly aids in speeding up the grouping process. This algorithm is simpler with regard to complexity. The time taken for executing the extreme case is o (n2t) since it contains 2 inner loops for going through n records and one loop for iteration. Identifying an objective function happens at the expense of huge computational cost. These benefits mentioned above make it flexible to tackle with continuous problems, clustering and classifications, and combinatorial optimization also.

Tuple partitioning section upholds two data structures. One is aimed toward the queue of buckets Q and the other for the sliced buckets SB. In the first section all the tuples are engaged in the bucket Q and the sliced bucket is divided into two buckets using MFA. This rule checks the intensity and attractiveness. Assume that there exist ‘n’ fireflies $x_{1} , \ldots ,x_{n}$ placed randomly in the space. The intensity for each firefly is linked with the objective function f(x). $I\alpha f(x)$. The fireflies attract each other on the basis of the intensity function. The firefly having the highest intensity will attract the other one. $I_{i} > I_{j} ,j = 1, \ldots ,n$. Attractiveness or the brightness of the firefly vary along with the distance between firefly i and firefly j. That is $r_{ij} = d(x_{i} ,x_{j} )$. The distance between any two fireflies i and j at $x_{i}$ and $x_{j}$ respectively is the Minkowsi distance measure. $X = \{ x_{1} , \ldots ,x_{n} \}\, \& Y = \{ y_{1} , \ldots ,y_{n} \}$

7 $$Minkowsi\;distance = \left( {\sum\limits_{i = 1}^{n} {|x_{i} - y_{i} |^{p} } } \right)^{1/p}$$

*P* = Minkowsi distance of order between the two points. This process continues until the bucket Q is empty. In each sliced bucket the diversity is checked. Finally the sliced table is reached. The subsequent steps define the proposed algorithm.


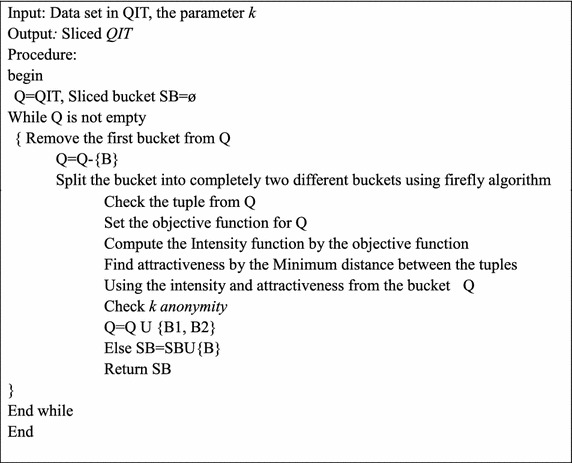


### Anonymised table

Attributes in the QIT and in each ST are sliced with the limiting factor of k-anonymity and *l*-diversity to ensure that there is no repetition of attribute values in each group. As soon as the tables are sliced off, the subsequent step is forming a SLAMSA anonymised group. In this approach, the SA in each group is shuffled and their linking with the QI attributes in QIT is done with a common group Id. This ensures that sensitive value of an individual that is involved in the QIT can be derived directly by an adversary with the probability of at most 1/*l*. A larger *l* results in stronger privacy. The process ends when there is no more remaining ungrouped SA or in the case when no new groups could be developed. If there is ungrouped transaction these are published in the form of a single group. It eliminates the overall complexity involved in privacy like membership disclosure, attribute and identity disclosure as this work adheres to the principle of both k-anonymity and *l*-diversity. Membership and identity disclosures are protected by k-anonymity and attribute disclosures are removed out by *l*-diversity.

## Performance analysis

The experiments were implemented in Java and carried out on a 3.3 GHz Intel Core processor with 20 GB hard disk and 3 GB RAM having Windows XP operating System.

The performance of the algorithm is tested over the datasets obtained from the Cleveland Clinic Foundation Heart disease and Hungarian Institute of Cardiology, which is available at http://archive.ics.uci.edu/ml/datasets/Heart+Disease. The data set comprises about 76 raw attributes that decide the probability with regard to the type of patient’s heart disease (e.g. class 1 or class 2 or class 3 or class 4). In this technical work, Age, sex, social security number, type of chest pain (Cp),blood pressure at rest (Rbp), serum Scestoral (Sc), blood sugar at fasting (Fbs), electrographic at rest (Restecg), maximum heart rate (Thalach), ST depression due to exercise related to rest (Old peak), exercise induced angina (Exang), slope of the peak exercise ST (Slope), number of major vessels (Ca), blood disorder (Thal), the predicted attribute (Class), which are significant for the ML researchers are taken into consideration. The attributes age, sex, and social security number are considered as QI and the other 12 attributes are considered as SA. These experiments have been performed to conceal the 12 SA attributes that decide over the probability of the type of the patient’s heart disease. The work proposed is realized on both the datasets that are mentioned above. The Cleveland Clinic Foundation Heart disease available at UCI machine learning repository has 303 instances. Since six patient records have more than 25 % of missing values, they are discarded from the dataset. After handling the missing values, the Cleveland dataset is reduced to 297. Similarly, the Hungarian Institute of Cardiology available at UCI machine learning repository has 294 instances and 34 patient records are discarded on account of the missing values. Thus the number of patient records which are taken into account by the proposed work is 557. The comparative evaluation is carried out between the system proposed and the existing MSB (Yang et al. 2008), SLOMS (Han et al. 2013) approaches.

### Utility

**Fig. 1:**
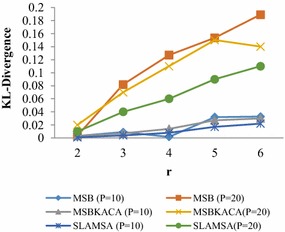
Reconstruction error versus r (m = 10)

**Fig. 2:**
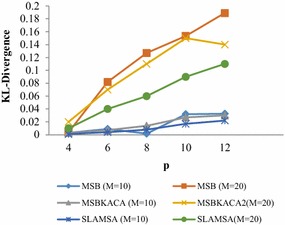
Reconstruction error versus p (r = 4)

**Fig. 3:**
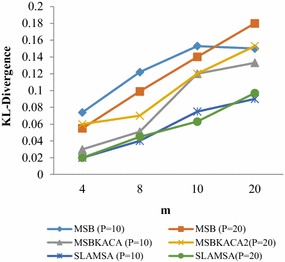
Reconstruction error versus m (r = 4)

In order to appraise the utilization of the published patient records, the determination of the reconstruction error is to be done for the queries discussed in utility requirements. The reconstruction error is actually measured by modifying p, m and r values in which p refers to the degree of privacy which is varied ranging from 4 to 20, m stands for the number of sensitive item which is randomly chosen between 3 and 12 and r refers to the number of QID which ranges between 2 and 4. 100 group-by queries are produced by randomly selecting q_1_, q_2_, q_3_, …, q_n_ and s_1_, s_2_, s_3_, …, s_m_. The average reconstruction error is determined and the utility is measured. The results are compared with MSB and SLOMS. The existing MSB and SLOMS techniques make use of generalization method which divides the dataset recursively as per QID values, till the privacy requirement does not permit any more splits. As the QID values within each group are generalized, it may have significant information loss. And those techniques which do not tackle with dimensionality are not appropriate for the preservation of multiple SA. On the contrary, this approach which exploits the advantage of anatomization, which releases the QID attributes, directly improves utility, meaning that information loss is also reduced. And enhanced slicing algorithm preserves correlations in a better manner between the attributes for better utility the SA satisfying *l*-diversity are taken into account. Figure 1 illustrates the result obtained for the dataset when parameter r is altered. The reconstruction error increases due to high dimensionality and thus limits utility. Then, r = 4 is fixed and the p value is changed. Figure 2 indicates that SLAMSA performs better than MSB and SLOMS approaches with respect to reconstruction error. As it is expected, while the privacy degree sees an increase, the reconstruction error also finds an increase in all the techniques and the utility is minimized. Thereafter, when the sensitive attributes is increased, reconstruction error is also increased. Figure 3 illustrates the result obtained for the value m. SLAMSA keeps up its performance in comparison with others as when m is greater. The more number of attributes in the query leads to more value matching. This limits the utility.

### Execution time analysis

The execution time of the proposed work is compared with the prevailing MSB and SLOMS approaches. The execution time of MSB and SLOMS is higher, as it needs to generalize the attributes in each dimension. But, the SLAMSA approach limits the execution time since this work obtains the advantages of anatomization along with the improved slicing approach. The anatomization approach eliminates generalization and reduces the execution time by direct release of the QI attributes. Advanced clustering algorithm (ACA) is proposed for the attributes partitioning into columns that reduce the necessity for the reassignment of the data point multiple number of times during each iteration. This can efficiently assist in enhancing the clustering speed and thereby reduce the complexity that is seen in the computation. But the algorithm complexity is high as it does not split the dataset further into several buckets. In order to minimize this problem and for finding the optimal size of buckets, tuple partitioning is formulated by MFA. It rapidly gets the equal size of buckets and then the partitioning is conducted in an effective way by the random behavior of the firefly. Additionally, in the proposed work, the single cluster group name is only utilized for referring to several datasets in the samples which also minimizes the complexity of the work.

**Fig. 4:**
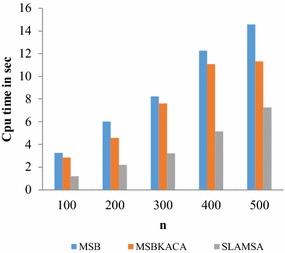
Execution time with respect to n (m = 4, l = 3)

In the first perspective, the execution time with regard to the proposed approach is increased slightly while the number of patient records (tuples) is raised. This is due to the fact that many tuples that require to be sliced and then anonymised. Figure 4 exhibits the execution time of the system by considering the number of patient records.

**Fig. 5:**
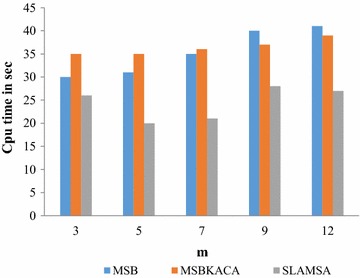
Execution time with respect to m (l = 3, n = 500)

In the second perspective, the execution time of the proposed work is measured by means of varying the number of SA from 3 to 12. The graphical result is shown in Fig. 5. When the SA is one, the execution time is same for all the cases. When the SA increases, the proposed techniques is required to process more SA and thereafter larger groups are needed to meet the *l*-diversity that again increases the running time. Experimental results show that SLAMSA keeps up its performance in comparison with other techniques when the SA increased.

This way, the proposed technique consumes lesser execution time and utility by a significant factor for any number of SA in a patients’ record.

## Conclusion

The important goal of this work is to preserve the privacy of the multiple SA and to improve the utility of the health care data. Slicing algorithm helps in preserving correlation and utility and anatomization minimizes the information loss. The advanced clustering algorithms exhibited its efficiency by minimizing the time and complexity. In addition, this work follows the principle of k-anonymity, *l*-diversity. This yields the means for the prevention of privacy threats like membership, identity and attributes disclosure. Also, this method can used to operate for any number of SA in an efficient manner.

In future, the slicing algorithm can be applied simultaneously to both QIT and ST to reduce the time further through increased processor speed and memory.

## Abbreviations

QI: quasi identifier
SA: sensitive attributes
ST: sensitive tables
QIT: quasi identifier table
MFA: metaheuristic firefly algorithm
PPDM: privacy preserving data mining
MSB: multiple sensitive bucketization
MFAMD: Minkowsi distance measure
